# Supplementary material for: Reading with deaf eyes: Automatic activation of speech-based phonology during word recognition is task dependent
Source: PLoS One. 2025 Aug 11;20(8):e0327142. doi: 10.1371/journal.pone.0327142 (PMC12338799; doi:10.1371/journal.pone.0327142)
Supplement: S1 Appendix — (DOCX) [file pone.0327142.s001.docx]

## **S1. Appendix A**

| Pseudo-homophone | English Word | Phonological Distractor | Semantic Distractor | Unrelated | Matched Word | Control Nonwords | Phonological Distractor | Semantic Distractor | Unrelated |
| --- | --- | --- | --- | --- | --- | --- | --- | --- | --- |
| **phly** | **fly** | sky | ant | crisps | **edge** | **adge** | wedge | target | stamp |
| **rhum** | **rum** | gum | wine | pen | **itch** | **atch** | stitch | cut (finger) | wall |
| **tize** | **ties** | pies | shoes | box | **guys** | **kuys** | dyes | ladies | cat |
| **nune** | **noon** | moon | watch | book | **wipe** | **jipe** | pipe | spray | clip |
| **kote** | **coat** | boat | shirt | swing | **claw** | **glaw** | saw | nails | curtains |
| **knoam** | **gnome** | foam | doll | pear | **queue** | **bueue** | shoe | crowd | flock |
| **skoar** | **score** | door | grade (exam/paper) | glove | **zebra** | **lebra** | bra | giraffe | jar |
| **wroes** | **rows** | bows | surf | glass | **alarm** | **olarm** | arm | police | peg |
| **beeze** | **bees** | peas | worm | hat | **whisk** | **chisk** | disc | bowl | bone (dog) |
| **koac** | **coke** | cloak | juice | ball | **crumb** | **grumb** | drum | drop | desk |
| **phell** | **fell** | well | run | brick | **eagle** | **aagle** | angle | crow | paint |
| **beaph** | **beef** | leaf | fish | car | **devil** | **tevil** | level | angel | apple |
| **phite** | **fight** | light | hug | spoon | **world** | **jorld** | gold | mars | bin |
| **wheap** | **weep** | sheep | smile | hop | **brush** | **prush** | flush | comb | cake |
| **wreek** | **reek** | beak | smoke | hands | **elves** | **alves** | shelves | fairy | pins |
| **hierze** | **hears** | beers | smells | drill | **robots** | **jobots** | dots | spock | jug |
| **knooce** | **noose** | goose | knot | phone | **pyjama** | **byjama** | llama | suit | poppies |
| **gneeze** | **knees** | cheese | feet | pool | **sydney** | **bydney** | knee | paris | rocket |
| **taughn** | **torn** | corn | cut (paper) | keys | **lizard** | **nizard** | wizard | frog | bells |
| **blede** | **bleed** | lead | cast | mug | **yacht** | **wacht** | cot | boat | chain |
| **trane** | **train** | rain | ship | plug | **queen** | **fueen** | bean | king | grass |
| **nale** | **nail** | tail | screw | belt | **lips** | **mips** | dips | nose | lemon |
| **stoan** | **stone** | cone | coal | knife | **razor** | **cazor** | laser | scissors | beach |
| **bair** | **bear** | chair | horse | heart | **glue** | **plue** | blue | pin | tin |
| **brooze** | **bruise** | fuse | grazed | snake | **toffee** | **doffee** | coffee | sweets | bat |
| **snale** | **snail** | whale | slug | fruit | **nappy** | **mappy** | happy | pants | lamp |
| **kirl** | **curl** | pearl | hair | lawn | **smog** | **swog** | dog | snow | folder |
| **fraim** | **frame** | flame | plaque (award) | purse | **cycle** | **gycle** | icicle | drive | lock |
