# Supplementary material for: Reading with deaf eyes: Automatic activation of speech-based phonology during word recognition is task dependent
Source: PLoS One. 2025 Aug 11;20(8):e0327142. doi: 10.1371/journal.pone.0327142 (PMC12338799; doi:10.1371/journal.pone.0327142)
Supplement: S2 Appendix — (DOCX) [file pone.0327142.s002.docx]

## **S2. Appendix B**

| Condition | Target | Distractor | Semantic | Unrelated |
| --- | --- | --- | --- | --- |
| homophone | **board** | bored | pins | gold |
| homophone | **break** | brake | rip | table |
| homophone | **flower** | flour | tree | file |
| homophone | **jeans** | genes | shirt | crisps |
| homophone | **hare** | hair | cow | lock |
| homophone | **leek** | leak | onion | printer |
| homophone | **moose** | mousse | bear | hat |
| homophone | **night** | knight | day | chocolate |
| homophone | **mussels** | muscles | lobster | desert |
| homophone | **poor** | pour | money | key |
| homophone | **rain** | rein | sun | worm |
| homophone | **rose** | rows | leaves | desk |
| homophone | **stake** | steak | bowarrow | envelope |
| homophone | **tail** | tale | monkey | comb |
| homophone | **tea** | tee | sugar | boy |
| homophone | **toe** | tow | fingers | bat |
| ortho_sim | **bell** | bull | alarm | jumper |
| ortho_sim | **beak** | bean | wing | path |
| ortho_sim | **beef** | beer | chicken | river |
| ortho_sim | **boat** | boot | anchor | mug |
| ortho_sim | **claw** | clap | fingernails | curtains |
| ortho_sim | **coat** | coal | skirt | swing |
| ortho_sim | **coke** | cone | juice | ball |
| ortho_sim | **cut** | cat | chop | lemon |
| ortho_sim | **food** | foot | milk | paper |
| ortho_sim | **frame** | flame | plaque | purse |
| ortho_sim | **glass** | grass | plate | house |
| ortho_sim | **gum** | gun | sweets | bee |
| ortho_sim | **ham** | hay | cheese | hook |
| ortho_sim | **hear** | head | smell | drill |
| ortho_sim | **pen** | peg | pencil | glasses |
| ortho_sim | **poke** | pole | hug | mouse |
| ortho_sim | **run** | rug | swim | baby |
| ortho_sim | **stone** | scone | sand | knife |
| ortho_sim | **tie** | tin | shoes | box |
| ortho_sim | **wire** | wine | wool | clip |
